# The emergence of bovine spongiform encephalopathy and related diseases.

**DOI:** 10.3201/eid0403.980311

**Published:** 1998

**Authors:** J Pattison

**Affiliations:** Medical School of University College London, United Kingdom.

## Abstract

Since 1986, approximately 170,000 cases of bovine spongiform encephalopathy (BSE) have occurred among approximately one million animals infected by contaminated feed in the United Kingdom. A ruminant feed ban in 1988 resulted in the rapid decline of the epidemic. Transmissible spongiform encephalopathies due to agents indistinguishable from BSE have appeared in small numbers of exotic zoo animals; a small outbreak among domestic cats is declining. Creutzfeldt-Jakob disease (CJD) has been intensively monitored since 1990 because of the risk BSE could pose to public health. In 1995, two adolescents in the United Kingdom died of CJD, and through the early part of 1996, other relatively young people had cases of what became known as new variant CJD, whose transmissible agent (indistinguishable from that of BSE) is responsible for 26 cases in the United Kingdom and one in France. Areas of concern include how many cases will appear in the future and whether or not use of human blood and blood products may cause a second cycle of human infections.


### The Emergence of Bovine Spongiform Encephalopathy and Related Diseases

**Sir John Pattison**
Medical School of University College London, London, United Kingdom

Since 1986, approximately 170,000 cases of bovine spongiform encephalopathy (BSE) have occurred among approximately one million animals infected by contaminated feed in the United Kingdom. A ruminant feed ban in 1988 resulted in the rapid decline of the epidemic. Transmissible spongiform encephalopathies due to agents indistinguishable from BSE have appeared in small numbers of exotic zoo animals; a small outbreak among domestic cats is declining. Creutzfeldt-Jakob disease (CJD) has been intensively monitored since 1990 because of the risk BSE could pose to public health. In 1995, two adolescents in the United Kingdom died of CJD, and through the early part of 1996, other relatively young people had cases of what became known as new variant CJD, whose transmissible agent (indistinguishable from that of BSE) is responsible for 26 cases in the United Kingdom and one in France. Areas of concern include how many cases will appear in the future and whether or not use of human blood and blood products may cause a second cycle of human infections.

Before the 1980s, a number of diseases of animals (scrapie, chronic wasting disease, and transmissible mink encephalopathy) and humans (Creutzfeldt-Jakob disease, Gerstmann-Straussler-Scheinker syndrome, and kuru), in spite of distinctive individual features, could be unified by the term transmissible spongiform encephalopathies (TSEs). In 1986, bovine spongiform encephalopathy (BSE) was first identified in indigenous cattle in the United Kingdom (1). A variety of clinical signs have been observed, but the three cardinal features of the disease are nervousness, heightened reactivity to external stimuli, and difficult movement, particularly of the hind limbs (2). Spongiform change is evident in the brain (1), and neuropathologic tests remain the mainstay of a BSE diagnosis. The disease was transmitted experimentally to mice (3) and cattle (4) by use of brain homogenates from cattle with clinical BSE; thus BSE has all the features that define classical TSEs.

### The BSE Epidemic

Some 1985 cases were diagnosed retrospectively; other cases occurring before 1986 probably went unnoticed. Since BSE was recognized, more than 170,000 cases were reported in the United Kingdom through the end of 1997. The epidemic curve, which peaked in 1992, is now in rapid decline (Table 1). Approximately two thirds of the dairy herds in the United Kingdom have had at least one case of BSE compared with only one sixth of the beef suckler herds. Furthermore, most of the affected suckler herds contained animals originating from dairy herds, which are fed differently.

Shortly after the recognition of BSE, epidemiologic studies indicated that the source of infection was the meat and bone meal used in concentrated cattle feed (5). Subsequently, in July 1988, ruminant protein in ruminant feed was banned. This ban immediately reduced the

Table 1. Annual incidence of bovine spongiform encephalopathy in the United Kingdom, 1985–1997

| Year | Number of cases |
|---|---|
| 1985 | 14 |
| 1986 | 60 |
| 1987 | 630 |
| 1988 | 2,184 |
| 1989 | 7,137 |
| 1990 | 14,181 |
| 1991 | 25,032 |
| 1992 | 36,682 |
| 1993 | 34,370 |
| 1994 | 23,945 |
| 1995 | 14,300 |
| 1996 | 8,016 |
| 1997 | 4,052 |

incidence of new infections, which began to be reflected in a diminution in the incidence of clinical cases 5 years later (the average incubation period) in 1993. Nevertheless, almost 36,000 cattle with BSE were born after the ruminant feed ban (a few as late as 1994), which indicates that the ban was not completely effective. Ruminant protein could be included in pig and poultry feed, and cross-contamination of cattle feed in the production mills and perhaps accidental exposure of cattle on the farm were possible until the feeding of mammalian protein to all farm animal species in the United Kingdom was prohibited in 1996.

The average age at which clinical BSE manifests itself is 4 to 5 years (6). Many animals in the national U.K. herd are slaughtered at significantly younger ages, and those infected with BSE would not have had a chance to develop the disease. Using methods developed for the retrospective analysis of the AIDS epidemic, Anderson and colleagues (7) calculated that approximately one million animals in the U.K. herd must have been infected to have produced 170,000 clinical cases of BSE. These same workers predicted the number of cases of BSE that would occur in 1996 and in subsequent years (Table 2). The calculations are based on a dominant feedborne source of infection; a small amount of cow-to-calf transmission was included because a long-term study, conducted by the U.K. Ministry of Agriculture, Fisheries and Food, indicated an increased incidence of BSE in calves born to mothers in the late stages of the incubation period of the disease (8). The results are compatible with a cow-to-calf transmission of approximately 10%, which in itself is not sufficient to perpetuate the BSE epidemic. The calculations predict a small number of cases and very few new infections by the beginning of the next decade. The predictions have been validated by the actual numbers in 1996 and 1997, which were 8,016 and 4,149, respectively (9).

### Infection in Other Animals

BSE has also been transmitted to exotic ruminants in zoos in the United Kingdom. Between 1986 and 1992, cases have occurred in bison, nyala, gemsbok, two species of oryx, greater kudu, and eland. These animals became infected by eating the same meat and bone meal-containing concentrated feed responsible for the disease in cattle. BSE infection in species other than ruminants was always considered possible. Careful watch was kept on the packs of hounds used for hunting in the United Kingdom because they are often fed carcasses unfit for human consumption. Spongiform encephalopathy has not occurred in dogs; however, in 1990, a case of spongiform encephalopathy was diagnosed in domestic cats; 81 additional cases in cats have occurred with a wide geographic spread throughout the United Kingdom. The true incidence is probably many times higher than observed because diagnosis is patchy and the disease was not statutorily notifiable until 1994. The annual incidence at the height of the outbreak was probably 10 to 15 cases per million cats (Wilesmith, pers. comm.). The most likely source of the infection was commercially produced cat food. In 1989, the pet food industry removed the dangerous bovine tissues, the specified bovine offal, before a statutory ban in 1990. The number of cases of feline spongiform encephalopathy (FSE) diagnosed in the United Kingdom has been declining since 1994 (1994, 16 cases; 1995, 6 cases; 1997, 6 cases) (Table 3). Only one cat, an adopted stray, was apparently born

Table 2. Predictions of new infections and cases of BSE[a] from 1996-2001[b]

| | New infections | | Cases | |
|---|---|---|---|---|
| Year | Expected value | 95% Prediction interval | Expected value | 95% Prediction interval |
| 1996 | 189 | (155-11,300) | 7,386 | (6,541-8,856) |
| 1997 | 95 | (63-236) | 4,111 | (3,006-7,664) |
| 1998 | 38 | (21-214) | 1,864 | (1,153-7,052) |
| 1999 | 12 | (5-162) | 682 | (388-5,909) |
| 2000 | 3 | (1-86) | 221 | (128-3,660) |
| 2001 | 1 | (0-33) | 72 | (45-1,592) |

[a]Bovine spongiform encephalopathy.
[b]Information extracted from (7).

Table 3. Number of cases of feline spongiform encephalopathy in the United Kingdom by year of diagnosis (MAFF, personal communication)

| Year | Number of cases |
|---|---|
| 1990 | 12 |
| 1991 | 12 |
| 1992 | 10 |
| 1993 | 11 |
| 1994 | 16 |
| 1995 | 8 |
| 1996 | 6 |
| 1997 | 6 |
| 1998[a] | 4 |

[a]To May 1, 1998.

after the ban on specified bovine offal in pet food. A TSE indistinguishable from BSE has also been found in puma, cheetah, ocelot, and a tiger in zoos in the United Kingdom between 1992 and 1995. These animals became infected as a result of being fed raw meat, which would have included bovine central nervous system, a practice which has now ceased.

### Human Disease and BSE

### Control Measures

The risk to human health from BSE was always recognized. The principal protective measure was the November 1989 ban on the use of certain specified bovine offal in human food. As with scraple, the tissues banned were those likely to contain the highest concentrations of the transmissible agent (brain, spinal cord, tonsil, spleen, thymus, and intestine of cattle older than 6 months of age). The intestine and thymus of calves was added to the list in 1994 when a long-term pathogenesis study in cattle by the Ministry of Agriculture, Fisheries and Food indicated that the transmissible agent could be found in the terminal ileum (it is assumed that the agent was present in Peyer's patches). In 1996 the whole head, other than the tongue, was formally banned because of concern about possible contamination with brain. Since the banned tissues now contain more than offal, the tissues are referred to as specified bovine material. During 1995, it became clear that spinal cord was not being completely removed from a small number of carcasses that were subsequently certified as fit for human consumption. Consequently, in December 1995 the U.K. government banned the use of bovine vertebral column for the production of mechanically recovered meat. In March 1996, when it became clear that human disease related to BSE was "probable" rather than "theoretical," the U.K. government introduced the over-30–month scheme, which allowed only animals under the age of 30 months to be used for human food, provided that all the banned specified bovine material had been removed. This added an extra margin of safety because cattle can be reasonably accurately aged by their dentition at 30 months and because BSE is relatively rare under the age of 30 months. Only 265 cases occurred in cattle younger than 30 months, and during 1997, the youngest animal with BSE was 37 months of age (Ministry of Agriculture, Fisheries and Food, pers. comm.). In the second half of 1997, the long-term pathogenesis experiment indicated that the transmissible agent of BSE could be recovered from the dorsal root ganglia of experimentally infected animals toward the end of the incubation period. Also, in one animal the agent was transmitted by intracerebral inoculation of mice with bovine bone marrow. Accordingly, in December 1997 the U.K. government introduced legislation to ban the sale of beef on the bone, even from animals under 30 months of age. Many in the United Kingdom thought that this regulation to prevent an extremely small risk of transmitting BSE in T-bone steaks and rib of beef was unnecessary. Nevertheless, the introduction of the specified bovine offal ban in 1989 and its subsequent refinements have ensured the safety of beef and beef products that now enter the human food chain in the United Kingdom. Even so, as a consequence of the emergence of new variant CJD, a worldwide ban on the sale of U.K. beef and beef products was introduced by the European Union in March 1996 and is still in force with the exception of a recent (March 1998) relaxation for certain herds in Northern Ireland.

### New Variant CJD

Clearly, the first measures to protect human health were introduced before any human disease could be related to BSE. To guard against the possible emergence of such disease (or diseases), the U.K. Department of Health set up a CJD Surveillance Unit in 1990. The purpose of the unit was to monitor the trends in incidence of CJD and any unusual features among cases. Concern was first focused on the 1995 cases of the third and fourth U.K. farmer since 1990 to be confirmed as having CJD. Statistically, the chances of four such cases occurring in 6 years in the United Kingdom were very small. However, the clinical features of the disease were typical of classical CJD, and collaboration between the CJD Surveillance Unit and other European countries indicated that farmers were overrepresented compared with CJD cases in countries with no BSE. Subsequently, classic CJD was confirmed in these farmers; no further cases have been diagnosed in U.K. farmers, and the significance of the high incidence in 1995 is diminishing.

The death in May 1995 of the first adolescent ever to be diagnosed with CJD in the United Kingdom was followed in October 1995 by the death of a second adolescent; by January 1996, three other young (29 years of age) persons became ill. Atypical pathologic results were beginning to be defined in these patients; and on March 8, 1996, eight cases of what came to be known as new variant CJD or variant CJD (vCJD) were reported to the Spongiform Encephalopathy Advisory Committee. The cases were distinguished by the relatively young age at which the symptoms started (10,11). That age range is now 16 years to 52 years. The duration of the illness is relatively long, averaging approximately 14 months as opposed to the 4 to 5 months in classic CJD. The early symptoms are often psychiatric, and it may be 6 or 7 months before any neurologic signs appear. The characteristic electroencephalogram pattern of sporadic CJD is not seen in vCJD, and pathologic results show florid plaques and extensive cerebellar involvement with multiple PrP deposits. As with BSE and FSE, the neuropathologic appearances are the mainstay of laboratory confirmation. Magnetic resonance imaging scanning and detection of 14-3-3 protein can be helpful. Early evidence indicates that the diagnosis can often be made from tonsil biopsies (12). Otherwise, diagnosis must depend upon brain biopsy or postmortem examination.

When on March 20, 1996, the U.K. government announced the existence of 10 cases of vCJD and the opinion of the Spongiform Encephalopathy Advisory Committee that these were probably related to BSE, three questions immediately arose. The first was, "Is there really any link with BSE?" Additional evidence emerged from the work of Collinge and his colleagues (13) on the analysis of the PrP fragments after protease digestion. The position of the three fragments and the relatively high concentrations of the di-glycosylated form indicated that vCJD was distinct from the previously recognized forms of CJD and that similarities existed between the cases of vCJD and BSE and FSE. In 1997, the first results were published from the classic strain typing experiments initiated during 1996 (14). The characteristics of material from cases of vCJD, in terms of incubation period and lesion profile in RIII mice, were identical to those from cases of BSE and FSE. These observations are confirmed now in C57 black mice. Thus, vCJD can now be regarded as human BSE in the same way that FSE is regarded as feline BSE. The second question was, "What is the route of transmission from cattle to humans?" So far we have no evidence, only a working hypothesis, that transmission was likely from inclusion in the human food chain of tissues that contain the highest concentration of the transmissible agent. The major differences in human exposure to these tissues would have occurred first when sick animals were banned from the human food chain in 1988 and again in 1989 when the specified bovine offals of otherwise healthy animals were removed from the human food chain.

Studies continue in an attempt to answer the third question, "How many vCJD cases will there be in the future?" So far, 26 cases have been diagnosed in the United Kingdom (Table 4) and 1 in France. Incidence has not increased since vCJD was first diagnosed in 1995. If instead of looking at the date of death one looks at the date of onset of the symptoms in the 26 patients, two new cases occurred on average every quarter since 1994. All cases have been methionine homozygotes at codon 129 of the PrP gene. In the general population approximately 40% have such a genotype; 10% are valine homozygotes, and 50% are heterozygotes. An analysis of classic sporadic CJD indicates that 80% of those cases are methionine homozygotes, 10% valine homozygotes, and 10% heterozygotes. It is perhaps not surprising, therefore, that the first cases of vCJD to be seen are methionine homozygotes.

Only one published analysis has predicted the number of future nvCJD cases after constraining the models used to the known and surmised facts at the time (15). The total number of future cases will depend critically on the average incubation period of vCJD. At present, we have no way of determining that; therefore, it remains too early to predict with any accuracy the total number of future cases. It remains possible that the outbreak of vCJD cannot be regarded as a single curve and that the small

Table 4. Creutzfeldt-Jakob disease in the United Kingdom

| | | Deaths of definite and probable cases | | | | | |
|---|---|---|---|---|---|---|---|
| Year | Referrals | Sporadic | Iatrogenic | Familial | GSS[a] | nvCJD[b] | Total |
| 1994 | 116 | 52 | 1 | 3 | 3 | 0 | 59 |
| 1995 | 86 | 34 | 4 | 2 | 3 | 3 | 46 |
| 1996 | 133 | 40 | 4 | 2 | 4 | 10 | 60 |
| 1997 | 152 | 42 | 6 | 3 | 0 | 10 | 61 |
| 1998[c] | 35 | 3 | 0 | 1 | 0 | 2 | 6 |

[a]Gerstmann-Straussler-Scheinker syndrome.
[b]New variant Creutzfeldt-Jakob disease.
[c]To Apr 30, 1998. Figures released by U.K. Department of Health Jun 1, 1998.

number of cases have occurred in persons who are extremely susceptible for unknown reasons.

### The Future

Much has happened already as a consequence of the emergence of BSE in U.K. cattle. The appropriate measures are in place to protect public health and end the BSE epidemic in cattle and other affected species. These measures are more rigorously enforced than ever before. It is difficult to see what could be done to make the BSE epidemic decline more rapidly than it already has, short of slaughtering the entire U.K. herd, which would be unnecessary and impractical. From now the question is likely to be how to withdraw some of the restrictions on U.K. beef and beef products. The exemptions are likely to be herd based (as is the case with Northern Ireland) or date based after the total ban on the use of meat and bone meal in the feed of any farm animals in the United Kingdom in 1996. In terms of the protection of public health, all the necessary measures are in place. Two further concerns remain and are actively under consideration: whether or not BSE exists in the sheep flocks and whether the cases of vCJD in the United Kingdom will be sufficient to generate concern about a second wave of transmission within the human population as a consequence of the use of blood and blood products. With respect to the former, the detection of sheep with scrapie-like diseases in the United Kingdom and the typing of strains from affected animals are being intensified. With respect to blood and blood products, some restrictions on the use of U.K. raw materials for the production of blood products are already in place, and a detailed risk assessment in relation to blood transfusion is awaited.

Sir John Pattison is a virologist and dean of the Medical School, University College London, and Chair, U.K. Spongiform Encephalopathy Advisory Committee.

### References

1. Wells GAH, Scott AC, Johnson CI, Gunning RF, Hancock RD, Jeffrey M, et al. A novel progressive spongiform encephalopathy in cattle. Vet Rec 1987;121:419-20.
2. Wilesmith JW, Hoinville LJ, Ryan JBM, Sayers AR. Bovine spongiform encephalopathy: aspects of the clinical picture and analyses of possible changes. Vet Rec 1992;130:197-201.
3. Fraser H, McConnell I, Wells GAH, Dawson M. Transmission of bovine spongiform encephalopathy to mice. Vet Rec 1988;123:472.
4. Dawson M, Wells GAH, Parker BNJ. Preliminary evidence of the experimental transmissibility of bovine spongiform encephalopathy to cattle. Vet Rec 1990;126:112-3.
5. Wilesmith JW, Wells GAH, Cranwell MP, Ryan JBM. Bovine spongiform encephalopathy: epidemiological studies. Vet Rec 1988;123:638-44.
6. Stekel DJ, Nowak MA, Southwood TRE. Prediction of future BSE spread. Nature 1996;381:119.
7. Anderson RM, Donnelly CA, Ferguson NM, Woolhouse MEJ, Watt CJ, Udy HJ, et al. Transmission dynamics and epidemiology of BSE in British cattle. Nature 1996;382:779-88.
8. Donnelly CA, Ghani AC, Ferguson NM, Wilesmith JW, Anderson RM. Analysis of the bovine spongiform encephalopathy study: evidence for direct maternal transmission. Appl Statist 1997;43:321-44.
9. Lawson C, Herd L, editors. BSE Enforcement Bulletin 1998. Ministry of Agriculture, Fisheries and Food; Bull. No. 20; p. 2.
10. Will RG, Ironside JW, Zeidler SN, Cousens SN, Estibeiro K, Alperovitch A, et al. A new variant of Creutzfeldt-Jakob disease in the UK. Lancet 1996;347:921-5.
11. Zeidler M, Stewart GE, Barraclough CR, Bateman DE, Bates D, Burn DJ, et al. New variant Creutzfeldt-Jakob disease: neurological features and diagnostic tests. Lancet 1997;350:903-7.
12. Hill AF, Zeidler M, Ironside J, Collinge J. Diagnosis of new variant Creutzfeldt-Jakob diseases by tonsil biopsy. Lancet 1997;349:99-100.
13. Collinge J, Sidle KCL, Meads J, Ironside J, Hill AF. Molecular analysis of prion strain variation and the aetiology of 'new variant' CJD. Nature 1996;383:685-90.
14. Bruce ME, Will RG, Ironside JW, McConnell I, Drummond D, Suttie A, et al. Transmissions to mice indicate that 'new variant' CJD is caused by the BSE agent. Nature 1997;389:498-501.
15. Cousens SN, Vynnycky E, Zeidler M, Will RG, Smith PG. Predicting the CJD epidemic in humans. Nature 1997;385:197-8.
